# On Compiling Structured CNFs to OBDDs

**DOI:** 10.1007/s00224-016-9715-z

**Published:** 2016-10-26

**Authors:** Simone Bova, Friedrich Slivovsky

**Affiliations:** Institute of Computer Graphics and Algorithms, TU Wien, 1040 Vienna, Austria

**Keywords:** Knowledge compilation, Ordered binary decision diagram, Conjunctive normal form, Expander graphs

## Abstract

We present new results on the size of OBDD representations of structurally characterized classes of CNF formulas. First, we prove that *variable convex* formulas (that is, formulas with incidence graphs that are convex with respect to the set of variables) have polynomial OBDD size. Second, we prove an exponential lower bound on the OBDD size of a family of CNF formulas with incidence graphs of bounded degree. We obtain the first result by identifying a simple sufficient condition—which we call the *few subterms* property—for a class of CNF formulas to have polynomial OBDD size, and show that variable convex formulas satisfy this condition. To prove the second result, we exploit the combinatorial properties of expander graphs; this approach allows us to establish an exponential lower bound on the OBDD size of formulas satisfying strong syntactic restrictions.

## Introduction

The goal of *knowledge compilation* is to succinctly represent propositional knowledge bases in a format that supports a number of queries in polynomial time [8]. Choosing a representation language generally involves a trade-off between succinctness and the range of queries that can be efficiently answered. In this paper, we study ordered binary decision diagram (OBDD) representations of propositional theories given as formulas in conjunctive normal form (CNF). Binary decision diagrams (also known as branching programs) and their variants are widely used and well-studied representation languages for Boolean functions [25]. OBDDs in particular enjoy properties, such as polynomial-time equivalence testing, that make them the data structure of choice for a range of applications.

**Fig. 1:**
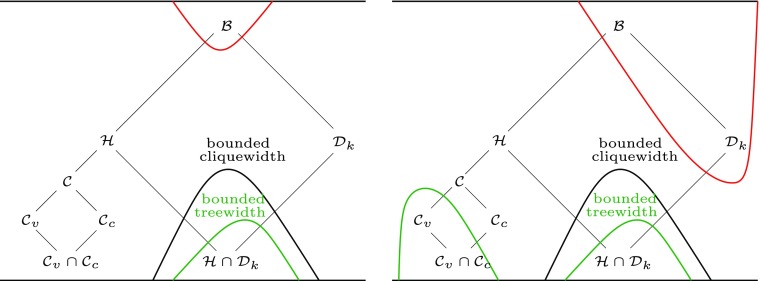
The diagram depicts a hierarchy of classes of bipartite graphs under the inclusion relation (thin edges). $\mathcal {B}$, $\mathcal {H}$, $\mathcal {D}_{k}$, $\mathcal {C}$, $\mathcal {C}_{v}$, and $\mathcal {C}_{c}$ denote, respectively, bipartite graphs, chordal bipartite graphs (corresponding to beta acyclic CNFs), bipartite graphs of degree at most *k* (*k* ≥ 3), convex graphs, *left* (variable) convex graphs, and *right* (clause) convex graphs. The class $\mathcal {C}_{v} \cap \mathcal {C}_{c}$ of biconvex graphs and the class $\mathcal {D}_{k}$ of bipartite graphs of degree at most *k* have unbounded clique-width. The class $\mathcal {H} \cap \mathcal {D}_{k}$ of chordal bipartite graphs of degree at most *k* has bounded treewidth. The *green* and *red curved lines* enclose, respectively, classes of incidence graphs whose CNFs have polynomial time OBDD compilation, and classes of incidence graphs whose CNFs have exponential size OBDD representations; the right hand picture shows the compilability frontier, updated in light of Results 1 and 2

The question of which classes of CNFs can be represented as (or *compiled into*, in the jargon of knowledge representation) OBDDs of polynomial size is largely unexplored [25, Chapter 4]. We approach this classification problem by considering *structurally* characterized CNF classes, more specifically, classes of CNF formulas defined in terms of properties of their *incidence graphs* (the incidence graph of a formula is the bipartite graph on clauses and variables where a variable is adjacent to the clauses it occurs in). Figure 1 depicts a hierarchy of well-studied bipartite graph classes as considered by Lozin and Rautenbach [20, Fig. 2]. This hierarchy is particularly well-suited for our classification project as it includes prominent cases such as beta acyclic CNFs [5] and bounded clique-width CNFs. When located within this hierarchy, the known bounds on the OBDD size of structural CNF classes leave a large gap (depicted *on the left* of Fig. 1):

- On the one hand, we have a polynomial upper bound on the OBDD size of bounded treewidth CNF classes proved recently by Razgon [23]. The corresponding graph classes are located at the bottom of the hierarchy.
- On the other hand, there is an exponential lower bound for the OBDD size of general CNFs, proved two decades ago by Devadas [9]. The corresponding graph class is not chordal bipartite, has unbounded degree and unbounded clique-width, and hence is located at the top of the hierarchy.

### **Contribution**

In this paper, we tighten this gap as illustrated *on the right* in Fig. 1. More specifically, we prove new bounds for two structural classes of CNFs.

### **Result 1**

CNF formulas with *variable convex* incidence graphs have polynomial OBDD size (Theorem 7).

Convexity is a property of bipartite graphs that has been extensively studied in the area of combinatorial optimization [14, 15, 24], and that can be detected in linear time [4, 19].

To prove Result 1, we identify a property of CNF classes—called the *few subterms property*—that is sufficient for polynomial-size compilability (Theorem 4), and then prove that CNFs with variable convex incidence graphs have this property (Lemma 6). The few subterms property naturally arises as a sufficient condition for polynomial size compilability when considering OBDD representations of CNF formulas (cf. Oztok and Darwiche’s recent work on *CV-width* [22], which explores a similar idea). Aside from its role in proving polynomial-size compilation for variable convex CNFs, the few subterms property can also be used to explain the (known) fact that classes of CNFs with incidence graphs of *bounded treewidth* have OBDD representations of polynomial size (Lemma 10), and as such offers a unifying perspective on these results. Both the result on variable convex CNFs and the result on bounded treewidth CNFs can be improved to polynomial *time* compilation by appealing to a stronger version of the few subterms property (Theorems 7 and 11).

In an attempt to push the few subterms property further, we adopt the language of *parameterized complexity* to formally capture the idea that CNFs “close” to a class with few subterms have “small” OBDD representations. More precisely, defining the *deletion distance* of a CNF from a CNF class as the number of its variables or clauses that have to be deleted in order for the resulting formula to be in the class, we prove that CNFs have fixed-parameter tractable OBDD size parameterized by the deletion distance from a CNF class with few subterms (Theorem 13). This result can again be improved to fixed-parameter *time* compilation under additional assumptions (Theorem 14), yielding for instance fixed-parameter tractable time compilation of CNFs into OBDDs parameterized by the *feedback vertex set* size (Corollary 15).

On the negative side, we prove that some structurally characterized classes of CNF formulas do not have small OBDD representations:

### **Result 2**

There is a class of CNF formulas with incidence graphs of bounded degree such that every formula *F* in this class has OBDD size at least 2^Ω(size(*F*))^ (Theorem 19).^1^

This substantially improves a $2^{\Omega (\sqrt {\text {\textsf {size}}(F)})}$ lower bound for the OBDD size of a class of CNFs by Devadas [9]. Moreover, we establish this bound for a class that satisfies strong syntactic restrictions: every clause contains exactly two positive literals and each variable occurs at most 3 times.

The heavy lifting in our proof of this result is done by a family of *expander graphs*. Expander graphs have found applications in many areas of mathematics and computer science [16, 21], including circuit and proof complexity [17]. In this paper, we show how they can be used to derive lower bounds for OBDDs.

### **Organization**

The remainder of this paper is organized as follows. In Section 2, we introduce basic notation and terminology. In Section 3, we prove that formulas with few subterms have polynomial OBDD size and show that variable-convex CNFs (as well as bounded treewidth CNFs) enjoy the few subterms property. Fixed-parameter tractable size and time compilability results based on the few subterms property are presented in Section 3.4. In Section 4, we prove a strongly exponential lower bound on the OBDD size of CNF formulas based on expander graphs. We conclude in Section 5.

## Preliminaries

### **Formulas**

Let *X* be a countable set of *variables*. A *literal* is a variable *x* or a negated variable ¬*x*. If *x* is a variable we let var(*x*) = var(¬*x*) = *x*. A *clause* is a finite set of literals. For a clause *c* we define var(*c*) = {var(*l*)∣*l* ∈ *c*}. If a clause contains a literal negated as well as unnegated it is *tautological*. A *conjunctive normal form (CNF)* is a finite set of non-tautological clauses. If *F* is a CNF formula we let $\text {\textsf {var}}(F) = \bigcup _{c \in F} \text {\textsf {var}}(c)$. The *size* of a clause *c* is |*c*|, and the *size* of a CNF *F* is $\text {\textsf {size}}(F) = {\sum }_{c \in F} |c|$. An *assignment* is a mapping *f* : *X* ^′^ → {0, 1}, where *X* ^′^ ⊆ *X*. We identify *f* with the set {¬*x*∣*x* ∈ *X* ^′^, *f*(*x*) = 0} ∪ {*x*∣*x* ∈ *X* ^′^, *f*(*x*) = 1}. An assignment *f* *satisfies* a clause *c* if *f* ∩ *c*≠*∅*; for a CNF *F*, we let *F*[*f*] denote the CNF containing the clauses in *F* not satisfied by *f*, restricted to variables in *X*∖var(*f*), that is, *F*[*f*] = {*c*∖{*x*, ¬*x*∣*x* ∈ var(*f*)}∣*c* ∈ *F*,*f* ∩ *c* = *∅*}; then, *f* *satisfies* *F* if *F*[*f*] = *∅*, that is, if it satisfies all clauses in *F*. If *F* is a CNF with var(*F*) = {*x*_1_,…,*x*_*n*_} we define the Boolean function *F*(*x*_1_,…,*x*_*n*_)*computed by* *F* as *F*(*b*_1_,…,*b*_*n*_) = 1 if and only if the assignment $f_{(b_{1}, \dots , b_{n})}: \text {\textsf {var}}(F) \rightarrow \{0, 1\}$ given by $f_{(b_{1}, \dots , b_{n})}(x_{i}) = b_{i}$ satisfies the CNF *F*.

### **Binary Decision Diagrams**

A *binary decision diagram (BDD)* *D* on variables {*x*_1_,…,*x*_*n*_} is a labelled directed acyclic graph satisfying the following conditions: *D* has at at most two vertices without outgoing edges, called *sinks* of *D*. Sinks of *D* are labelled with 0 or 1; if there are exactly two sinks, one is labelled with 0 and the other is labelled with 1. Moreover, *D* has exactly one vertex without incoming edges, called the *source* of *D*. Each non-sink node of *D* is labelled by a variable *x*_*i*_, and has exactly two outgoing edges, one labelled 0 and the other labelled 1. Each node *v* of *D* represents a Boolean function *F*_*v*_ = *F*_*v*_(*x*_1_,…,*x*_*n*_) in the following way. Let (*b*_1_,…,*b*_*n*_) ∈ {0, 1}^*n*^ and let *w* be a node labelled with *x*_*i*_. We say that (*b*_1_,…,*b*_*n*_)*activates* an outgoing edge of *w* labelled with *b* ∈ {0, 1} if *b*_*i*_ = *b*. Since (*b*_1_,…,*b*_*n*_) activates exactly one outgoing edge of each non-sink node, there is a unique sink that can be reached from *v* along edges activated by (*b*_1_,…,*b*_*n*_). We let *F*_*v*_(*b*_1_,…,*b*_*n*_) = *b*, where *b* ∈ {0, 1} is the label of this sink. The function *computed by* *D* is *F*_*s*_, where *s* denotes the (unique) source node of *D*. The *size* of a BDD is the number of its nodes.

An *ordering* *σ* of a set {*x*_1_,…,*x*_*n*_} is a total order on {*x*_1_,…,*x*_*n*_}. If *σ* is an ordering of {*x*_1_,…,*x*_*n*_} we let var(*σ*) = {*x*_1_,…,*x*_*n*_}. Let *σ* be the ordering of {*x*_1_,…,*x*_*n*_} given by $x_{i_{1}}<x_{i_{2}}< {\cdots } <x_{i_{n}}$. For every integer 0 < *j* ≤ *n*, the *length* *j* *prefix* of *σ* is the ordering of $\{x_{i_{1}},\ldots ,x_{i_{j}}\}$ given by $x_{i_{1}}< {\cdots } <x_{i_{j}}$. A *prefix* of *σ* is a length *j* prefix of *σ* for some integer 0 < *j* ≤ *n*. For orderings $\sigma =x_{i_{1}}< {\cdots } <x_{i_{n}}$ of {*x*_1_,…,*x*_*n*_} and $\rho =y_{i_{1}}< {\cdots } <y_{i_{m}}$ of {*y*_1_,…,*y*_*m*_}, we let *σ* *ρ* denote the ordering of {*x*_1_,…,*x*_*n*_, *y*_1_,…,*y*_*m*_} given by $x_{i_{1}}< {\cdots } <x_{i_{n}}<y_{i_{1}}< {\cdots } <y_{i_{m}}$. Let *D* be a BDD on variables {*x*_1_,…,*x*_*n*_} and let $\sigma = x_{i_{1}} < {\cdots } < x_{i_{n}}$ be an ordering of {*x*_1_,…,*x*_*n*_}. The BDD *D* is a *σ*-ordered binary decision diagram (*σ*-OBDD) if *x*_*i*_ < *x*_*j*_ (with respect to *σ*) whenever *D* contains an edge from a node labelled with *x*_*i*_ to a node labelled with *x*_*j*_. A BDD *D* on variables {*x*_1_,…,*x*_*n*_} is an *ordered binary decision diagram (OBDD)* if there is an ordering *σ* of {*x*_1_,…,*x*_*n*_} such that *D* is a *σ*-OBDD. For a Boolean function *F* = *F*(*x*_1_,…,*x*_*n*_), the *OBDD size* of *F* is the size of the smallest OBDD on {*x*_1_,…,*x*_*n*_} computing *F*.

We say that a class $\mathcal {F}$ of CNFs has *polynomial-time compilation into OBDDs* if there is a polynomial-time algorithm that, given a CNF $F \in \mathcal {F}$, returns an OBDD computing the same Boolean function as *F*. We say that a class $\mathcal {F}$ of CNFs *has polynomial size compilation into OBDDs* if there exists a polynomial $p \colon \mathbb {N} \to \mathbb {N}$ such that, for all CNFs $F \in \mathcal {F}$, there exists an OBDD of size at most *p*(size(*F*)) that computes the same function as *F*.

### **Graphs**

For standard graph theoretic terminology, see [10]. Let *G* = (*V*,*E*) be a graph. The *(open) neighborhood* of *W* in *G*, in symbols neigh(*W*,*G*), is defined by

$$\text{\textsf{neigh}}(W,G) =\{ v \in V \setminus W \mid \text{there exists}\; w \in W\; \text{such that}\; vw \in E\}.$$

We freely use neigh(*v*,*G*) as a shorthand for neigh({*v*},*G*), and we write neigh(*W*) instead of neigh(*W*,*G*) if the graph *G* is clear from the context. A graph *G* = (*V*,*E*) is *bipartite* if its vertex set *V* can be partitioned into two blocks *V* ^′^ and *V* ^″^ such that, for every edge *v* *w* ∈ *E*, we either have *v* ∈ *V* ^′^ and *w* ∈ *V* ^″^, or *v* ∈ *V* ^″^ and *w* ∈ *V* ^′^. In this case we may write *G* = (*V* ^′^, *V* ^″^, *E*). The *incidence* graph of a CNF *F*, in symbols inc(*F*), is the bipartite graph (var(*F*),*F*,*E*) such that *v* *c* ∈ *E* if and only if *v* ∈ var(*F*), *c* ∈ *F*, and *v* ∈ var(*c*); that is, the blocks are the variables and clauses of *F*, and a variable is adjacent to a clause if and only if the variable occurs in the clause.

A bipartite graph *G* = (*V*,*W*,*E*) is *left convex* if there exists an ordering *σ* of *V* such that the following holds: if *wv* and *w* *v* ^′^ are edges of *G* and *v* < *v* ^″^ < *v* ^′^ (with respect to the ordering *σ*) then *w* *v* ^″^ is an edge of *G*. The ordering *σ* is said to *witness* left convexity of *G*. A CNF *F* is *variable convex* if inc(*F*) = (var(*F*),*F*,*E*) is left convex.

For an integer *d*, a CNF *F* has *degree* *d* if inc(*F*) has degree at most *d*. A class $\mathcal {F}$ of CNFs has *bounded degree* if there exists an integer *d* such that every CNF in $\mathcal {F}$ has degree *d*.

## Polynomial Time Compilability

In this section, we introduce the *few subterms* property, a sufficient condition for a class of CNFs to admit polynomial size compilation into OBDDs (Section 3.1). We prove that the classes of variable convex CNFs and bounded treewidth CNFs have the few subterms property (Sections 3.2 and 3.3). Finally, we establish fixed-parameter tractable size and time OBDD compilation results for CNFs, where the parameter is the deletion distance to a few subterms CNF class (Section 3.4).

### The Few Subterms Property

#### **Definition 1** (Subterm width)

Let *F* be a CNF formula and let *V*⊆var(*F*). The set of *V*- subterms of *F* is defined

$$\text{\textsf{st}}(F,V) = \{ F[f] \mid f \colon V \to \{0, 1\} \}.$$

Given an ordering *σ* of var(*F*), the *subterm* width of *F* with respect to *σ* is

$$\text{\textsf{stw}}(F,\sigma) =\max \{ |\text{\textsf{st}}(F,\text{\textsf{var}}(\pi))| \mid \pi\text{ is a prefix of }\sigma \}.$$

The *subterm width* of *F* is the minimum subterm width of *F* with respect to an ordering of var(*F*).

We now state a few simple properties of subterms that will be used throughout the paper. Let *V* be a set of variables and let *F* be a CNF.

1. Trivially, |st(*F*,*V*)| ≤ 2^|*V*|^.
2. Each *V*-subterm of *F* is the restriction of a subset of *F*, so |st(*F*,*V*)| ≤ 2^|*F*|^.
3. If *F* = *F* ^′^∪*F* ^″^ then *F*[*f*] = *F* ^′^[*f*]∪*F* ^″^[*f*] for every variable assignment *f* : *V*→{0, 1}, so |st(*F*,*V*)| ≤ |st(*F* ^′^, *V*)|⋅|st(*F* ^″^, *V*)|.

#### **Definition 2** (Subterm Bound)

Let $\mathcal {F}$ be a class of CNF formulas. A function $b \colon \mathbb {N} \to \mathbb {N}$ is a *subterm bound* of $\mathcal {F}$ if, for all $F \in \mathcal {F}$, the subterm width of *F* is bounded from above by *b*(size(*F*)). Let $b \colon \mathbb {N} \to \mathbb {N}$ be a subterm bound of $\mathcal {F}$, let $F \in \mathcal {F}$, and let *σ* be an ordering of var(*F*). We call *σ* a *witness* of the subterm bound *b* with respect to *F* if stw(*F*,*σ*) ≤ *b*(size(*F*)).

#### **Definition 3** (Few Subterms)

A class $\mathcal {F}$ of CNF formulas has *few subterms* if it has a polynomial subterm bound $p \colon \mathbb {N} \to \mathbb {N}$; if, in addition, for all $F \in \mathcal {F}$, an ordering *σ* of var(*F*) witnessing *p* with respect to *F* can be computed in polynomial time, $\mathcal {F}$ is said to have *constructive few subterms*.

The few subterms property naturally presents itself as a sufficient condition for a polynomial size construction of OBDDs from CNFs.

#### **Theorem 4**

*There exists an algorithm that, given a CNF F and an ordering σ of* var(*F*), *returns a σ-OBDD for F of size at most* |var(*F*)|⋅stw(*F, σ*) *in time polynomial in* |var(*F*)| *and* stw(*F, σ*).

#### *Proof*

Let *F* be a CNF and *σ* = *x*_1_<⋯ < *x*_*n*_ be an ordering of var(*F*). The algorithm computes a *σ*-OBDD *D* for *F* as follows.

At step *i* = 1, create the source of *D*, labelled by *F*, at level 0 of the diagram; if *∅* ∈ *F* (respectively, *F* = *∅*), then identify the source with the 0-sink (respectively, 1-sink) of the diagram, otherwise make the source an *x*_1_-node.

At step *i*+1 for *i* = 1,…,*n*−1, let *v*_1_,…,*v*_*l*_ be the *x*_*i*_-nodes at level *i*−1 of the diagram, respectively labelled *F*_1_,…,*F*_*l*_. For *j* = 1,…,*l* and *b* = 0, 1, compute *F*_*j*_[*x*_*i*_ = *b*], where *x*_*i*_ = *b* denotes the assignment *f*:{*x*_*i*_}→{0, 1} mapping *x*_*i*_ to *b*. If *F*_*j*_[*x*_*i*_ = *b*] is equal to some label of an *x*_*i*+1_-node *v* already created at level *i*, then direct the *b*-edge leaving the *x*_*i*_-node labelled *F*_*j*_ to *v*; otherwise, create a new *x*_*i*+1_-node *v* at level *i*, labelled *F*_*j*_[*x*_*i*_ = *b*], and direct the *b*-edge leaving the *x*_*i*_-node labelled *F*_*j*_ to *v*. If *∅* ∈ *F*_*j*_[*x*_*i*_ = *b*], then identify *v* with the 0-sink of *D*, and if *∅* = *F*_*j*_[*x*_*i*_ = *b*], then identify *v* with the 1-sink of *D*.

At termination, the diagram obtained computes *F* and respects *σ*. We analyze the runtime. At step *i*+1 (0 ≤ *i* < *n*), the nodes created at level *i* are labelled by CNFs of the form *F*[*f*], where *f* ranges over all assignments of {*x*_1_,…,*x*_*i*_} not falsifying *F*; that is, these nodes correspond exactly to the {*x*_1_,…,*x*_*i*_}-subterms st(*F*,{*x*_1_,…,*x*_*i*_}) of *F* not containing the empty clause, whose number is bounded above by stw(*F*,*σ*). As level *i* is processed in time bounded above by its size times the size of level *i*−1, and |var(*F*)| levels are processed, the diagram *D* has size at most |var(*F*)|⋅stw(*F*,*σ*) and is constructed in time bounded above by a polynomial in |var(*F*)| and stw(*F*,*σ*). □

#### **Corollary 5**

*A class of CNFs with the constructive few subterms property admits polynomial time compilation into OBDDs.*

#### *Proof*

Let $\mathcal {F}$ be a class of CNF formulas with constructive few subterms and let $p \colon \mathbb {N} \to \mathbb {N}$ be a polynomial subterm bound of $\mathcal {F}$. The algorithm, given a CNF *F*, computes in polynomial time an ordering of var(*F*) witnessing *p* with respect to *F*, and invokes the algorithm of Theorem 4, which runs in time polynomial in |var(*F*)| and stw(*F*,*σ*). Since stw(*F*,*σ*) ≤ *p*(size(*F*)) the overall runtime is polynomial in size(*F*). □

### Variable Convex CNF formulas

In this section, we prove that the class of variable convex CNFs has the constructive few subterms property (Lemma 6), and hence admits polynomial time compilation into OBDDs (Theorem 7); as a special case, CNFs whose incidence graphs are cographs admit polynomial time compilation into OBDDs (Example 8).

#### **Lemma 6**

*The class* $\mathcal {F}$ *of variable convex CNFs has the constructive few subterms property.*

#### *Proof*

Let $F \in \mathcal {F}$, so that inc(*F*) is left convex, and let *σ* be an ordering of var(*F*) witnessing the left convexity of inc(*F*). Let *π* be any prefix of *σ*. Call a clause *c* ∈ *F* *π*-*active* in *F* if var(*c*)∩var(*π*)≠*∅* and var(*c*)∩(var(*F*)∖var(*π*))≠*∅*. Let *A* denote the set of *π*-active clauses of *F*. For all *c* ∈ *A*, let var _*π*_(*c*) = var(*c*) ∩ var(*π*).

#### *Claim*

Let *c*,*c* ^′^∈*A*. Then, var _*π*_(*c*)⊆var _*π*_(*c* ^′^) or var _*π*_(*c* ^′^)⊆var _*π*_(*c*).

#### *Proof of Claim*

Let *c*,*c* ^′^∈*A*. Assume for a contradiction that the statement does not hold, that is, there exist variables *v*,*v* ^′^∈var(*π*), *v*≠*v* ^′^, such that *v* ∈ var _*π*_(*c*)∖var _*π*_(*c* ^′^) and *v* ^′^∈var _*π*_(*c* ^′^)∖var _*π*_(*c*). Assume that *σ*(*v*) < *σ*(*v* ^′^); the other case is symmetric. Since *c* is *π*-active, by definition there exists a variable *w* ∈ var(*F*)∖var(*π*) such that *w* ∈ var(*c*). It follows that *σ*(*v* ^′^) < *σ*(*w*). Therefore, we have *σ*(*v*) < *σ*(*v* ^′^) < *σ*(*w*), where *v*,*w* ∈ var(*c*) and *v* ^′^∉var(*c*), contradicting the fact that *σ* witnesses the left convexity of inc(*F*). □

We now argue that there is a function *g* with domain *A* such that the image of *A* under *g* contains the set {*A*[*f*] | *f* does not satisfy *A*} of terms induced by assignments not satisfying *A*. Let *L* = {*x*, ¬*x* | *x* ∈ var(*π*)} denote the set of literals associated with variables in var(*π*). The function *g* is defined as follows. For *c* ∈ *A*, we let

$$g(c) = \{ c^{\prime} \setminus L \:|\: c^{\prime} \in A, c^{\prime} \cap L \subseteq c \cap L \}.$$

Let *f* : var(*π*)→{0, 1} be an assignment that does not satisfy *A*. Let *c* ∈ *A* be a clause not satisfied by *f* such that var _*π*_(*c*) is maximal with respect to inclusion. We claim that *g*(*c*) = *A*[*f*]. To see this, let *c* ^′^∈*A* be an arbitrary clause. It follows from the claim proved above that either $\text {\textsf {var}}_{\pi }(c) \subsetneq \text {\textsf {var}}_{\pi }(c^{\prime })$ or var _*π*_(*c* ^′^)⊆var _*π*_(*c*). In the first case, *c* ^′^ is satisfied by choice of *c*. In the second case, *c* ^′^ is not satisfied by *f* if and only if *c* ^′^ ∩ *L*⊆*c* ∩ *L*. The formula *A*[*f*] is precisely the set of clauses in *A* not satisfied by *f*, restricted to variables not in var(*π*), so *g*(*c*) = *A*[*f*] as claimed. Taking into account that an assignment might satisfy *A*, this implies

$$|\text{\textsf{st}}(A, \text{\textsf{var}}(\pi))| \leq |A| + 1 \leq \text{\textsf{size}}(F) + 1.$$

Let *A* ^′^ = {*c* ∈ *F* | var(*c*)⊆var(*π*)} and *A* ^″^ = {*c* ∈ *F* | var(*c*) ∩ var(*π*) = *∅*}, so that *F* = *A*∪*A* ^′^∪*A* ^″^. For every assignment *f* : var(*π*)→{0, 1} we have *A* ^″^[*f*] = *A* ^″^ and either *A* ^′^[*f*] = *∅* or *A* ^′^[*f*] = {*∅*}, so the number of subterms of *F* under assignments to var(*π*) is bounded as

$$|\text{\textsf{st}}(F, \text{\textsf{var}}(\pi))| \leq 2 \cdot (\text{\textsf{size}}(F) + 1).$$

This proves that the class of variable convex CNFs has few subterms. Moreover, an ordering witnessing the left convexity of inc(*F*) can be computed in polynomial (even linear) time [4, 19], so the class of variable convex CNFs even has the constructive few subterms property. □

#### **Theorem 7**

*The class of variable convex CNF formulas has polynomial time compilation into OBDDs.*

#### *Proof*

Immediate from Corollary 5 and Lemma 6. □

#### *Example 8* (*Bipartite Cographs*)

Let *F* be a CNF such that inc(*F*) is a cograph. Note that inc(*F*) is a complete bipartite graph. Indeed, cographs are characterized as graphs of clique-width at most 2 [7], and it is readily verified that if a bipartite graph has clique-width at most 2, then it is a complete bipartite graph. A complete bipartite graph is trivially left convex. Then Theorem 7 implies that CNFs whose incidence graphs are cographs have polynomial time compilation into OBDDs.

### Bounded Treewidth CNF Formulas

In this section, we prove that if a class of CNFs has *bounded treewidth*, then it has the constructive few subterms property (Lemma 10), and hence admits polynomial time compilation into OBDDs (Theorem 11).

Let *G* be a graph. A *tree decomposition* of *G* is a triple $\mathcal {T} = (T, \chi , r)$, where *T* = (*V*(*T*),*E*(*T*)) is a tree rooted at *r* and *χ* : *V*(*T*)→2^*V*(*G*)^ is a labeling of the vertices of *T* by subsets of *V*(*G*) (called *bags*) such that

1. $\bigcup _{t \in V(T)} \chi (t) = V(G)$,
2. for each edge *u* *v* ∈ *E*(*G*), there is a node *t* ∈ *V*(*T*) with {*u*,*v*}⊆*χ*(*t*), and
3. for each vertex *v* ∈ *V*(*G*), the set of nodes *t* with *v* ∈ *χ*(*t*) forms a connected subtree of *T*.

The *width* of a tree decomposition (*T*,*χ*,*r*) is the size of a largest bag *χ*(*t*) minus 1. The *treewidth* of *G* is the minimum width of a tree decomposition of *G*. The *pathwidth* of *G* is the minimum width of a tree decomposition (*T*,*χ*,*r*) such that *T* is a path.

Let *F* be a CNF. We say that inc(*F*) = (var(*F*),*F*,*E*) has treewidth (respectively, pathwidth) *k* if the graph (var(*F*)∪*F*,*E*) has treewidth (respectively, pathwidth) *k*. We identify the pathwidth (respectively, treewidth) of a CNF with the pathwidth (respectively, treewidth) of its incidence graph.

Let *F* be a CNF formula. If inc(*F*) has pathwidth *k*, then an ordering *σ* of var(*F*) is called a *forget ordering* for *F* if, with respect to an arbitrary linearization of some path decomposition of width *k* for inc(*F*), if the first bag containing *v* is less than or equal to the first bag containing *v* ^′^ whenever *σ*(*v*) < *σ*(*v* ^′^). A proof of the following lemma already appears, in essence, in works by Ferrara et al. [12, Theorem 2.1] and Razgon [23, Lemma 5].

#### **Lemma 9**

*Let F be a CNF of pathwidth k* − 1, *and let σ be a forget ordering for F. Then* stw(*F, σ*) ≤ 2^*k*+1^.

#### *Proof*

Let *F* be a CNF such that inc(*F*) has pathwidth *k*−1, let *σ* be a forget ordering for *F*, and let *π* be any prefix of *σ*.

Let *v* be the last variable in var(*π*) relative to the ordering *σ*, and let *B* be the first bag (in the linearization of *P*) that contains *v*. A clause *c* ∈ *F* is called *π*-*active* in *F* if var(*c*) ∩ var(*π*) ≠ *∅* and var(*c*) ∩ (var(*F*) ∖ var(*π*)) ≠ *∅*. Let ac(*F*,var(*π*)) denote the CNF containing the *π*-active clauses of *F*. Let

$$\begin{array}{@{}rcl@{}} C^{\prime} &=& \text{\textsf{ac}}(F,\text{\textsf{var}}(\pi)) \cap B,\\ C^{\prime\prime} &=& \{ c \in \text{\textsf{ac}}(F,\text{\textsf{var}}(\pi)) \mid c \in B^{\prime}\; \text{only if}\; B^{\prime}>B\; \text{in} P \}; \end{array} $$

in words, *C* ^′^ contains *π*-active clauses in the bag *B*, and *C* ^″^ contains *π*-active clauses occurring only in bags strictly larger than *B* in the total order of *P*. Clearly, *C* ^′^ ∩ *C* ^″^ = *∅*.

#### *Claim*

ac(*F*,var(*π*)) = *C* ^′^∪*C* ^″^.

#### *Proof of Claim*

First observe that a *π*-active clause *c* cannot occur only in bags strictly smaller than *B* in the total order of *P*. For otherwise, since var(*c*)∩(var(*F*)∖var(*π*))≠*∅*, let *v* ^′^∈var(*c*)∩(var(*F*)∖var(*π*)); if *B* ^′^ is the first bag that contains *v* ^′^, then *B* ≤ *B* ^′^ (by the choice of *v*), hence *v* ^′^ is not contained in any bag strictly smaller than *B*, and the edge *c* *v* ^′^ is not witnessed in *P*, a contradiction.

Thus *π*-active clauses either occur in *B* (including the case where they occur in *B* and in bags smaller or larger than *B* in *P*), or occur only in bags strictly larger than *B* in *P*. Thus, ac(*F*, var(*π*)) ⊆ *C* ^′^∪*C* ^″^; the other inclusion holds by definition. □

From the claim, we get

$$|\text{\textsf{st}}(\text{\textsf{ac}}(F,\text{\textsf{var}}(\pi)), \text{\textsf{var}}(\pi))| \leq |\text{\textsf{st}}(C^{\prime},\text{\textsf{var}}(\pi))| \cdot |\text{\textsf{st}}(C^{\prime\prime},\text{\textsf{var}}(\pi))|\text{;}$$

thus, it suffices to bound above the size of the two sets on the right so that the product of the individual bounds is at most 2^*k*^. Let *k* ^′^ = |*C* ^′^|. Obviously, we have $|\text {\textsf {st}}(C^{\prime },\text {\textsf {var}}(\pi ))|\leq 2^{k^{\prime }}$. Let $V^{\prime }=\bigcup _{c \in C^{\prime \prime }} \text {\textsf {var}}(c) \cap \text {\textsf {var}}(\pi )$ and let *k* ^″^ = |*V* ^′^|.

#### *Claim*

*V* ^′^ ⊆ *B*.

#### *Proof of Claim*

Let *c* be a *π*-active clause occurring only in bags strictly larger than *B* in *P*. Let *v* ^′^∈var(*c*) ∩ var(*π*). By the choice of *v* and the properties of the forget ordering *σ*, it holds that the first bag containing *v* ^′^ is less than or equal to *B*. Since *B* is the first bag that contains *v*, it holds that *v* ^′^∈*B* by the properties of *P* (the edge *c* *v* ^′^ is witnessed in a bag strictly larger than *B* in *P*). □

#### *Claim*

$|\text {\textsf {st}}(C^{\prime \prime },\text {\textsf {var}}(\pi ))|\leq 2^{k^{\prime \prime }}$.

#### *Proof of Claim*

Define an equivalence relation on var(*π*)-assignments as follows: For all *f*,*f* ^′^:var(*π*)→{0, 1}, *f*≡*f* ^′^ if and only if, for all *v* ∈ *V* ^′^, *f*(*v*) = *f* ^′^(*v*). Since |*V* ^′^| = *k* ^″^, the equivalence relation has $2^{k^{\prime \prime }}$ many equivalence classes. Moreover, if *f*≡*f* ^′^, then *C* ^″^[*f*] = *C* ^″^[*f* ^′^], because var(*C* ^″^) ⊆ *V* ^′^. The claim follows. □

Since *C* ^′^, *V* ^′^ ⊆ *B* and *C* ^′^ ∩ *V* ^′^ = *∅*, it holds that *k* ^′^ + *k* ^″^ = |*C* ^′^|+|*V* ^′^| ≤ |*B*| ≤ *k*. Hence,

$$|\text{\textsf{st}}(\text{\textsf{ac}}(F,\text{\textsf{var}}(\pi)), \text{\textsf{var}}(\pi))| \leq 2^{k^{\prime}} \cdot 2^{k^{\prime\prime}}=2^{k^{\prime}+k^{\prime\prime}} \leq 2^{k}\text{.}$$

Note that *F* is the disjoint union of ac(*F*,var(*π*)), clauses *D* ^′^ ⊆ *F* whose variables are all in var(*π*), and clauses *D* ^″^ ⊆ *F* whose variables are all outside var(*π*). Also, st(*D* ^′^, var(*π*))⊆{*∅*,{*∅*}}, and st(*D* ^″^, var(*π*)) = {*D* ^″^}. It follows that

$$\begin{array}{@{}rcl@{}} |\text{\textsf{st}}(F,\text{\textsf{var}}(\pi))| &\leq& |\text{\textsf{st}}(\text{\textsf{ac}}(F,\text{\textsf{var}}(\pi)), \text{\textsf{var}}(\pi))| \cdot |\text{\textsf{st}}(D^{\prime},\text{\textsf{var}}(\pi))| \cdot |\text{\textsf{st}}(D^{\prime\prime},\text{\textsf{var}}(\pi))| \\ &\leq& 2^{k+1}, \end{array} $$

and the statement is proved. □

#### **Lemma 10**

*Let* $\mathcal {F}$ *be a class of CNFs of bounded treewidth. Then* $\mathcal {F}$ *has the constructive few subterms property.*

#### *Proof*

Let *c*−1 be the treewidth bound of $\mathcal {F}$ and let $F \in \mathcal {F}$, so that the treewidth of inc(*F*) is at most *c*−1. We can compute a width *c*−1 tree decomposition of inc(*F*) in linear time *O*(size(*F*)) [3]. From this decomposition, we can compute a path decomposition of inc(*F*) of width at most (*c*−1)⋅ log|var(*F*) ∪ *F*| ≤ *c*⋅ log|var(*F*) ∪ *F*|−1 [2, Corollary 24] and a corresponding forget ordering of var(*F*) in polynomial time. By Lemma 9, the subterm width of *F* with respect to *σ* is at most 2^*c*⋅log|var(*F*)∪*F*|^ = |var(*F*)∪*F*|^*c*^ ≤ *O*(size(*F*)^*c*^). Thus $\mathcal {F}$ has a polynomial subterm bound, and a witnessing ordering *σ* can be computed for each $F \in \mathcal {F}$ in polynomial time. We conclude that $\mathcal {F}$ has the constructive few subterms property. □

#### **Theorem 11**

*Let* $\mathcal {F}$ *be a class of CNFs of bounded treewidth. Then,* $\mathcal {F}$ *has polynomial time compilation into OBDDs.*

#### *Proof*

Immediate from Lemma 10 and Corollary 5. □

### Almost Few Subterms

In this section, we use the language of *parameterized complexity* to formalize the observation that CNF classes “close” to CNF classes with few subterms have “small” OBDD representations [11, 13].

Let *F* be a CNF and *D* a set of variables and clauses of *F*. Let *E* be the formula obtained by deleting *D* from *F*, that is,

$$E=\{ c \setminus \{ l \in c \mid \text{\textsf{var}}(l) \in D \} \mid c \in F \setminus D \}\text{;}$$

we call *D* a *deletion set* of *F* with respect to *E*.

The following lemma shows that adding a few variables and clauses does not increase the subterm width of a formula too much.

#### **Lemma 12**

*Let F and E be CNFs such that D is a deletion set of F with respect to E. Let π be an ordering of* var(*E*) *and let σ be an ordering of* var(*F*) ∩ *D. Then* stw(*F, σπ*) ≤ 2^*k*^ ⋅ stw(*E, π*), *where k* = |*D*|.

#### *Proof*

Let *V* = *D* ∩ var(*F*) and *C* = *D* ∩ *F*, and let *k* ^′^ = |*V*| and *k* ^″^ = |*C*|. Let *ρ* be a prefix of *σ* *π* and *X* = var(*ρ*). From *F* = *C*∪(*F*∖*C*) we get

1 $$ |\text{\textsf{st}}(F, X)| \leq |\text{\textsf{st}}(C, X)|\: |\text{\textsf{st}}(F \setminus C, X)|.  $$

By definition,

$$\text{\textsf{st}}(F \setminus C, X) = \{(F \setminus C)[f]\:|\:f \in \{0, 1\}^{X} \}.$$

Splitting the assignments *f* into two parts, we can write this as

2 $$ \text{\textsf{st}}(F \setminus C, X) = \{(F \setminus C)[f^{\prime}][f^{\prime\prime}]\:|\:f^{\prime} \in \{0, 1\}^{V \cap X}, f^{\prime\prime} \in \{0, 1\}^{X \setminus V} \}.  $$

Let *f* ^′^∈{0, 1}^*V* ∩ *X*^ be an assignment. The formula *E* is obtained from *F*∖*C* by deleting variables in *V*. It follows that (*F*∖*C*)[*f* ^′^]⊆*E* and thus

$$(F \setminus C)[f^{\prime}][f^{\prime\prime}] \subseteq E[f^{\prime\prime}]$$

for any assignment *f* ^″^∈{0, 1}^*X*∖*V*^. This yields

3 $$ |\{(F \setminus C)[f^{\prime}][f^{\prime\prime}]\:|\:f^{\prime\prime} \in \{0, 1\}^{X \setminus V} \}| \leq |\{E[f^{\prime\prime}]\:|\:f^{\prime\prime} \in \{0, 1\}^{X \setminus V} \}|,  $$

and the right hand side of this inequality corresponds to |st(*E*,*X*∖*V*)|. Combining this with (2), we obtain

$$\begin{array}{@{}rcl@{}} |\text{\textsf{st}}(F \setminus C, X)| &=& |\{ (F \setminus C)[f^{\prime}][f^{\prime\prime}]\:|\:f^{\prime} \in \{0, 1\}^{V \cap X}, f^{\prime\prime} \in \{0, 1\}^{X \setminus V} \}| \\ &\leq& 2^{k^{\prime}} |\{E[f^{\prime\prime}]\:|\:f^{\prime\prime} \in \{0, 1\}^{X \setminus V} \}| = 2^{k^{\prime}} \cdot |\text{\textsf{st}}(E, X \setminus V)| \\ &\leq& 2^{k^{\prime}}\cdot \text{\textsf{stw}}(E, \pi). \end{array} $$

Inserting into (1), we get

$$\begin{array}{@{}rcl@{}} |\text{\textsf{st}}(F, X)| \leq |\text{\textsf{st}}(C, X)|\cdot |\text{\textsf{st}}(F \setminus C, X)| \leq 2^{k^{\prime\prime}} \cdot 2^{k^{\prime}}\cdot \text{\textsf{stw}}(E, \pi) = 2^{k} \cdot \text{\textsf{stw}}(E, \pi), \end{array} $$

and the lemma is proved. □

In this section, the standard of efficiency we appeal to comes from the framework of *parameterized complexity* [11, 13]. The parameter we consider is defined as follows. Let $\mathcal {F}$ be a class of CNF formulas. We say that $\mathcal {F}$ is *closed under variable and clause deletion* if $E \in \mathcal {F}$ whenever *E* is obtained by deleting variables or clauses from $F \in \mathcal {F}$. Let $\mathcal {F}$ be a CNF class closed under variable and clause deletion. The $\mathcal {F}$-deletion distance of *F* is the minimum size of a deletion set of *F* from any $E \in \mathcal {F}$. An $\mathcal {F}$-deletion set of *F* is a deletion set of *F* with respect to some $E \in \mathcal {F}$.

Let $\mathcal {F}$ be a class of CNF formulas with few subterms closed under variable and clause deletion. We say that CNFs have *fixed-parameter tractable OBDD size*, parameterized by $\mathcal {F}$-deletion distance, if there is a computable function $f:\mathbb {N} \rightarrow \mathbb {N}$ and a polynomial $p: \mathbb {N} \rightarrow \mathbb {N}$ such that a CNF *F* with $\mathcal {F}$-deletion distance *k* has OBDD size at most *f*(*k*) *p*(size(*F*)).

#### **Theorem 13**

*Let* $\mathcal {F}$ *be a class of CNF formulas with few subterms closed under variable and clause deletion. CNFs have fixed-parameter tractable OBDD size, parameterized by* $\mathcal {F}$ *-deletion distance.*

#### *Proof*

Let $\mathcal {F}$ be a class of CNF formulas with few subterms closed under variable and clause deletion. Since $\mathcal {F}$ has few subterms, it has a polynomial subterm bound $p: \mathbb {N} \rightarrow \mathbb {N}$. Let *k* be the $\mathcal {F}$-deletion distance of *F*. Let $E \in \mathcal {F}$ be a formula such that the deletion distance of *F* from *E* is *k*, and let *D* a deletion set of *F* with respect to *E*. Let *π* be an ordering of var(*E*) witnessing *p* for *E*, and let *σ* be an ordering of var(*F*) ∩ *D*. By Lemma 12, the subterm width of *F* with respect to *ρ* = *σ* *π* is at most 2^*k*^ *p*(size(*E*)), so by Theorem 4 there is a *ρ*-OBDD for *F* of size at most 2^*k*^ *p*(size(*E*)) |var(*F*)|. □

The requirement that $\mathcal {F}$ be closed under variable and clause deletion ensures that the deletion distance from $\mathcal {F}$ is defined for every CNF. For our purposes, this can be assumed without loss of generality, as it is readily verified that if $\mathcal {F}$ has few subterms with polynomial subterm bound $p: \mathbb {N} \rightarrow \mathbb {N}$, then the closure of $\mathcal {F}$ under variable and clause deletion has few subterms with the same polynomial subterm bound.

Analogously, we say that CNFs have *fixed-parameter tractable time computable OBDDs* (respectively, $\mathcal {F}$-deletion sets), parameterized by $\mathcal {F}$-deletion distance, if an OBDD (respectively, a $\mathcal {F}$-deletion set) for a given CNF *F* of $\mathcal {F}$-deletion distance *k* is computable in time *f*(*k*) *p*(size(*F*)).

#### **Theorem 14**

*Let* $\mathcal {F}$ *be a class of CNFs closed under variable and clause deletion satisfying the following:*

- $\mathcal {F}$ *has the constructive few subterms property*.
- *CNFs have fixed-parameter tractable time computable* $\mathcal {F}$ *-deletion sets, parameterized by* $\mathcal {F}$ *-deletion distance*.

*Then CNFs have fixed-parameter tractable time computable OBDDs, parameterized by* $\mathcal {F}$ *-deletion distance.*

#### *Proof*

Given an input formula *F*, the algorithm first computes a smallest $\mathcal {F}$-deletion set *D* of *F*. Let *E* be the formula obtained from *F* by deleting *D*. The algorithm computes a variable ordering *π* of *E* witnessing a polynomial subterm bound $p: \mathbb {N} \rightarrow \mathbb {N}$ of $\mathcal {F}$. Since $\mathcal {F}$ has the constructive few subterms property, this can be done in polynomial time. Next, the algorithm chooses an arbitrary ordering *σ* of var(*F*) ∩ *D*. By Lemma 12 we have stw(*F*,*σ* *π*) ≤ 2^|*D*|^ stw(*E*,*π*) ≤ 2^*k*^ *p*(size(*E*)), where *k* is the $\mathcal {F}$-deletion distance of *F*. Invoking the algorithm of Theorem 4, our algorithm computes and returns an OBDD for *F* in time polynomial in 2^*k*^ *p*(size(*E*)) |var(*F*)|. Since size(*E*) ≤ size(*F*) there is a polynomial $q: \mathbb {N} \rightarrow \mathbb {N}$ (independent of *F*) such that the last expression is bounded by 2^*k*^ *q*(size(*F*)). □

#### **Corollary 15** (**Feedback Vertex Set**)

*Let* $\mathcal {F}$ *be the class of formulas whose incidence graphs are forests. CNFs have fixed-parameter tractable time computable OBDDs parameterized by* $\mathcal {F}$ *-deletion distance.*

#### *Proof*

Given a graph *G* = (*V*,*E*), a set *D*⊆*V* is called a *feedback vertex set* of *G* if the graph *G*∖*D* is a forest; here, *G*∖*D* is the graph (*V*∖*D*,*E* ^′^) such that *v* *w* ∈ *E* ^′^ if and only if *v* *w* ∈ *E* and *v*,*w* ∈ *V*∖*D*. For any CNF *F*, a subset *D* of its variables and clauses is a feedback vertex set of the incidence graph inc(*F*) if and only if it is a $\mathcal {F}$-deletion set, so a smallest feedback vertex set of inc(*F*) is a smallest $\mathcal {F}$-deletion set. There is a fixed-parameter tractable algorithm that, given a graph *G* and a parameter *k*, computes a feedback vertex set *D* of *G* such that |*D*| ≤ *k* or reports that no such set exists [6]. It follows that there is a fixed-parameter tractable algorithm, parameterized by the $\mathcal {F}$-deletion distance, for computing a smallest $\mathcal {F}$-deletion set of an input CNF. Moreover, the incidence graphs of formulas in $\mathcal {F}$ have treewidth 1, so $\mathcal {F}$ has the constructive few subterms property by Lemma 10. Clearly, $\mathcal {F}$ is closed under variable and clause deletion. Hence, applying Theorem 14, we conclude that CNFs have fixed-parameter tractable time computable OBDDs parameterized by $\mathcal {F}$-deletion distance. □

## Polynomial Size Incompilability

In this section, we introduce the *subfunction width* of a graph CNF, to which the OBDD size of the graph CNF is exponentially related (Section 4.1), and prove that *expander graphs* yield classes of graph CNFs of *bounded degree* with linear subfunction width, thus obtaining an exponential lower bound on the OBDD size for graph CNFs in such classes (Section 4.2).

### Many Subfunctions

In this section, we introduce the *subfunction width* of a graph CNF (Definition 16), and prove that the OBDD size of a graph CNF is bounded below by an exponential function of its subfunction width (Theorem 17).

A *graph CNF* is a CNF *F* such that *F* = {{*u*,*v*}∣*u* *v* ∈ *E*} for some graph *G* = (*V*,*E*) without isolated vertices.

#### **Definition 16** (Subfunction Width)

Let *F* be a graph CNF. Let *σ* be an ordering of var(*F*) and let *π* be a prefix of *σ*. We say that a subset {*c*_1_,…,*c*_*e*_} of clauses in *F* is *subfunction productive* relative to *π* if there exist {*a*_1_,…,*a*_*e*_}⊆var(*π*) and {*u*_1_,…,*u*_*e*_}⊆var(*F*)∖var(*π*) such that for all *i*,*j* ∈ {1,…,*e*}, *i*≠*j*, and all *c* ∈ *F*,

- *c*_*i*_ = {*a*_*i*_, *u*_*i*_};
- *c*≠{*a*_*i*_, *a*_*j*_} and *c*≠{*a*_*i*_, *u*_*j*_}.

The *subfunction width* of *F*, in symbols sfw(*F*), is defined by

$$\text{\textsf{sfw}}(F)=\min_{\sigma} \max_{\pi} \{ |M| \mid M\; \text{is subfunction productive relative to}\; \pi \},$$

where *σ* ranges over all orderings of var(*F*) and *π* ranges over all prefixes of *σ*.

Intuitively, in the graph *G* underlying the graph CNF *F* in Definition 16, there is a matching of the form *a*_*i*_ *u*_*i*_ with *a*_*i*_∈var(*π*) and *u*_*i*_∈var(*F*)∖var(*π*), *i* ∈ {1,…,*e*}; such a matching is “almost” induced, in that *G* can contain edges of the form *u*_*i*_ *u*_*j*_, but no edges of the form *a*_*i*_ *a*_*j*_ or *a*_*i*_ *u*_*j*_, *i*,*j* ∈ {1,…,*e*}, *i*≠*j*.

#### **Theorem 17**

*Let F be a graph CNF. The OBDD size of F is at least* 2^sfw(*F*)^.

#### *Proof*

Let *F* be a graph CNF. Let *D* be any OBDD computing *F*, let *σ* be the ordering of var(*F*) respected by *D*, and let *π* be a prefix of *σ* such that {*c*_1_,…,*c*_*e*_}⊆*F* is subfunction productive relative to *σ* and *π* and *e* ≥ sfw(*F*). Let {*a*_1_,…,*a*_*e*_}⊆var(*π*) and {*u*_1_,…,*u*_*e*_}⊆var(*F*)∖var(*π*) be as in Definition 16, so that in particular *c*_*i*_ = {*a*_*i*_, *u*_*i*_}, *i* ∈ {1,…,*e*}. Let

4 $$ L=\{ f \colon \text{\textsf{var}}(\pi) \to \{0, 1\} \mid f(v)=1\; \text{for all}\; v \not\in \{a_{1},\ldots,a_{e}\} \}; $$

in words, *L* is the set containing, for each assignment of {*a*_1_,…,*a*_*e*_}, its extension to var(*π*) that sends all variables in var(*π*)∖{*a*_1_,…,*a*_*e*_} to 1.

#### *Claim*

Let *f* ∈ *L* and let *c* ∈ *F* be such that *c*⊆var(*π*). Then, *f* satisfies *c*.

#### *Proof of Claim*

Otherwise, since *F* is a graph CNF and by (4) the only variables sent to 0 by *f* are in {*a*_1_,…,*a*_*e*_}, it is the case that *c* = {*a*_*i*_, *a*_*j*_} for some *i*,*j* ∈ {1,…,*e*}, *i*≠*j*, which is impossible by the second item in Definition 16. □

#### *Claim*

Let *f* and *g* be distinct assignments in *L*. Then, *f* and *g* lead to different nodes in *D*.

#### *Proof of Claim*

Let *f* and *g* be distinct assignments in *L*. By the previous claim, *f* and *g* satisfy each clause in *F* whose variables are contained in var(*π*). Thus, the computation paths activated by *f* and *g* in *D* lead to some nodes in *D* distinct from the 0-sink of *D*.

Since *f* and *g* are distinct assignments in *L*, they differ on at least one variable in {*a*_1_,…,*a*_*e*_}; say without loss of generality that *f*(*a*_1_) = 0≠1 = *g*(*a*_1_). Let *h* : var(*F*)∖var(*π*)→{0, 1} be such that *h*(*v*) = 0 if and only if *v* = *u*_1_. We show that that *f*∪*h* does not satisfy *F*, but *g*∪*h* satisfies *F*; it follows that *f* and *g* lead to different nodes in *D*.

Clearly, *f*∪*h* does not satisfy *F*, because by Definition 16 the clause *c*_1_ = {*a*_1_, *u*_1_} is in *F*, and by construction *f*(*a*_1_) = *h*(*u*_1_) = 0. We show that *g*∪*h* satisfies *F*.

Let *c* ∈ *F*. If *c*⊆var(*π*), then *g* satisfies *c* by the previous claim. If *c*⊆var(*F*)∖var(*π*), then *h* satisfies *c*, because *c* contains two distinct variables, hence at least one of its variables differs from *u*_1_ and is assigned to 1 by *h*. Otherwise, *c* ∩ var(*π*)≠*∅* and *c*∩(var(*F*)∖var(*π*))≠*∅*. If *c* contains a variable in var(*F*)∖var(*π*) distinct from *u*_1_, then *h* satisfies *c*. Otherwise, *c* = {*a*,*u*_1_} for some *a* ∈ var(*π*). In this case, if *a* ∈ {*a*_1_,…,*a*_*e*_}, then *a* = *a*_1_ by Definition 16, and *g* satisfies *c* via *g*(*a*_1_) = 1. Else, *a* ∈ var(*π*)∖{*a*_1_,…,*a*_*e*_} and by definition of *L* we have *g*(*a*) = 1, so that again *g* satisfies *c*. □

It is readily observed that |*L*|=2^*e*^. Then, by the above claims, the computation paths activated by the assignments in *L* lead to 2^*e*^ different nodes in *D*. We observed that *e* ≥ sfw(*F*). Then *D* has size at least 2^sfw(*F*)^. It follows that the OBDD size of *F* is at least 2^sfw(*F*)^. □

### Bounded Degree

In this section, we use the existence of a family of *expander graphs* to obtain a class of graph CNFs with linear subfunction width (Lemma 18), thus obtaining an exponential lower bound on the OBDD size of a class of CNFs of *bounded degree* (Theorem 19).

Let *n* and *d* be positive integers, *d* ≥ 3, and let *𝜖*<1 be a positive real. A graph *G* = (*V*,*E*) is a (*n*,*d*,*𝜖*)-*expander* if *G* has *n* vertices, degree at most *d*, and for all subsets *W*⊆*V* such that |*W*| ≤ *n*/2, the inequality

5 $$ |\text{\textsf{neigh}}(W)| \geq \epsilon|W| $$

holds. It is known that for all integers *d* ≥ 3, there exists a real 0 < *𝜖*, and a sequence

6 $$ \{ G_{i} \mid i \in \mathbb{N} \} $$

such that *G*_*i*_ = (*V*_*i*_, *E*_*i*_) is an (*n*_*i*_, *d*,*𝜖*)-expander ($i \in \mathbb {N}$), and *n*_*i*_ tends to infinity as *i* tends to infinity [1, Section 9.2].

#### **Lemma 18**

*Let F be a graph CNF whose underlying graph is an (n,d,𝜖)-expander, where n ≥ 2, 𝜖>0, and d ≥ 3. Then*

$$\text{\textsf{sfw}}(F) \geq \frac{\epsilon }{16d}\: n\text{.} $$

#### *Proof*

Let *σ* be any ordering of var(*F*) and let *π* be the length ⌊*n*/2⌋ prefix of *σ*.

#### *Claim*

There exists a subset {*c*_1_,…,*c*_*l*_} of clauses of *F*, subfunction productive relative to *π*, such that $l \geq \frac {\epsilon }{16d}\, n$.

#### *Proof of Claim*

We will construct a sequence (*a*_1_, *b*_1_),…,(*a*_*l*_, *b*_*l*_) of pairs (*a*_*i*_, *b*_*i*_)∈var(*π*)×(var(*F*)∖var(*π*)) of vertices such that *a*_*i*_∉neigh(*a*_*j*_), and such that {*a*_*i*_, *b*_*j*_} ∈ *F* if and only if *i* = *j*, for 1 ≤ *i*, *j* ≤ *l*. Letting *c*_*i*_ = {*a*_*i*_, *b*_*i*_} for 1 ≤ *i* ≤ *l*, this yields a set {*c*_1_,…,*c*_*l*_} of clauses that are subfunction productive relative to *π*. Assume we have chosen a (possibly empty) sequence (*a*_1_, *b*_1_),…,(*a*_*j*_, *b*_*j*_) of such pairs. For a vertex *v* in the underlying graph of *F*, let *N*[*v*] = {*v*}∪neigh(*v*) denote its solid neighborhood. Let $V = \bigcup _{i=1}^{j} (N[a_{i}] \cup N[b_{i}])$ and *A* = var(*π*)∖*V*. Then |*A*| ≤ *n*/2 and we can use the expansion property (5) to conclude that |neigh(*A*)| ≥ *𝜖*|*A*|. Let *B* = neigh(*A*)∖*V*. If both *A* and *B* are nonempty we pick (*a*_*j*+1_, *b*_*j*+1_)∈*A*×*B* so that *a*_*j*+1_ *b*_*j*+1_ is an edge. We have *A*⊆var(*π*) as well as *B*⊆var(*F*)∖(*A*∪*V*)⊆var(*F*)∖var(*π*), so (*a*_*j*+1_, *b*_*j*+1_)∈var(*π*)×(var(*F*)∖var(*π*)). By construction, {*a*_*j*+1_, *b*_*j*+1_} is a clause in *F*; moreover, *a*_*i*_∉neigh(*b*_*j*+1_) as well as *b*_*i*_∉neigh(*a*_*j*+1_), for 1 ≤ *i* ≤ *j*. We conclude that the sequence (*a*_1_, *b*_1_),…,(*a*_*j*+1_, *b*_*j*+1_) has the desired properties. Otherwise, if either of the sets *A* or *B* is empty, we stop.

We now give a lower bound on the length *l* of a sequence constructed in this manner. Let (*a*_1_, *b*_1_),…,(*a*_*j*_, *b*_*j*_) be such that one of the sets *A* and *B* as defined in the previous paragraph is empty, so that *j* = *l*. Since the degree of the underlying graph is bounded by *d*, we have |*V*| ≤ 2*d* *j* and |*A*| ≥ ⌊*n*/2⌋−2*d* *j*. If *A* is empty, we must have 2*d* *j* ≥ ⌊*n*/2⌋ and thus

7 $$ j \geq \left\lfloor \frac{n}{2} \right\rfloor\: \frac{1}{2d} \geq \frac{n-1}{4d} \geq \frac{n}{8d}, $$

where the last inequality follows from *n* ≥ 2. Now suppose *B* is empty. We have |*B*| ≥ *𝜖*|*A*|−|*V*|, so

$$0 \geq \epsilon (\lfloor n/2 \rfloor - 2dj) - 2dj = \epsilon (\lfloor n/2 \rfloor) - 2dj (1+\epsilon). $$

From this, we get

8 $$ j \geq \frac{\epsilon (n - 1)}{4d (1+\epsilon)} \geq \frac{\epsilon (n - 1)}{8d} \geq \frac{\epsilon n}{16d}. $$

Here, the last inequality again follows from *n* ≥ 2. Recalling that *𝜖*<1 and taking the minimum of the bounds in (7) and (8), we obtain the lower bound stated in the claim. □

The lemma is an immediate consequence of the above claim. □

#### **Theorem 19**

*There exist a class* $\mathcal {F}$ *of CNF formulas and a constant c>0 such that, for every* $F \in \mathcal {F}$, *the OBDD size of F is at least* 2^*c* ⋅ size(*F*)^. *In fact,* $\mathcal {F}$ *is a class of read 3 times, monotone, 2-CNF formulas.*

#### *Proof*

Let $\mathcal {G}=\{ G_{i} \mid i \in \mathbb {N} \}$ be a family of graphs as in (6), so that for all $i \in \mathbb {N}$ the graph *G*_*i*_ = (*V*_*i*_, *E*_*i*_) is a (*n*_*i*_, *d*,*𝜖*)-expander (*n*_*i*_ ≥ 2, *d* = 3, *𝜖*>0) and *n*_*i*_→*∞* as *i*→*∞*. Using the expansion property, it is readily verified that each graph in $\mathcal {G}$ is connected; in particular, it does not have isolated vertices. Therefore $\mathcal {F}=\{ E_{i} \colon i \in \mathbb {N} \}$ is a class of graph CNFs; in fact, a class of read 3 times, monotone, 2-CNF formulas. By Lemma 18 each $F \in \mathcal {F}$ satisfies

$$\text{\textsf{sfw}}(F) \geq \frac{\epsilon}{16d} \: |\text{\textsf{var}}(F)|.$$

Since the underlying graph of *F* has degree at most *d* and |var(*F*)| vertices, the formula *F* contains at most *d* |var(*F*)| clauses (each variable occurs in at most *d* clauses), and each clause contains at most 2 literals. That is, 2*d*|var(*F*)| ≥ size *F*, and thus

$$\text{\textsf{sfw}}(F) \geq \frac{\epsilon}{16d}\:|\text{\textsf{var}}(F)| = \frac{\epsilon}{32d^{2}} \:2d|\text{\textsf{var}}(F)| \geq \frac{\epsilon}{32d^{2}}\: \text{\textsf{size}}{F}.$$

Setting *c* = *𝜖* / 32*d* ^2^, it follows from Theorem 17 that the OBDD size of *F* is at least 2^*c*⋅|size*F*|^. □

## Conclusion

In closing, we briefly explain why completing the classification task laid out in this paper (and thus closing the gap depicted in Fig. 1) seems to require new ideas.

On the one hand, our upper bound for variable convex CNFs appears to push the few subterms property to its limits – natural variable orderings cannot be used to witness few subterms for (clause) convex CNFs and CNF classes of bounded clique-width. On the other hand, our lower bound technique based on expander graphs essentially requires bounded degree, but the candidate classes for improving lower bounds in our hierarchy, bounded clique-width CNFs and beta acyclic CNFs, have unbounded degree. In both cases, imposing a degree bound leads to classes of bounded treewidth [18] and thus polynomial bounds on the size of OBDD representations.

## Footnotes

1 Here, size(*F*) denotes the number of variable occurrences in the formula *F*.
